# Ancestry inferences from DNA testing results: The problem of sociogenetic essentialism

**DOI:** 10.1007/s40656-025-00670-w

**Published:** 2025-05-16

**Authors:** Kostas Kampourakis, Michal Fux

**Affiliations:** 1 Section of Biology and IUFE, University of Geneva, Geneva, Switzerland; 2 Brain and Cognitive Science Department, Massachusetts Institute of Technology, Cambridge, MA USA

**Keywords:** Psychological essentialism, Social essentialism, Genetic essentialism, Ancestry informative markers, DNA ancestry testing, Race

## Abstract

Millions of people have now taken DNA ancestry tests, with many of them looking for information about their origins or even their ethnic identity. However, what these tests can only do is allow for a probabilistic estimate of a person’s similarity to a reference group. This is often based on research in population genetics that study human genetic variation by identifying ancestry informative markers, that is, DNA markers that are found more often in one population rather than others. Whereas these markers are not the criteria for membership in a group, they can serve as indicia for it. However, a confusion of indicia for criteria can emerge supported by a particular form of intuitive thinking, psychological essentialism. It consists of a set of interrelated beliefs: (a) Particular categories distinguish between fundamentally different kinds of people; (b) The boundaries that separate these categories are strict and absolute; (c) These categories have internal homogeneity and differ fundamentally from one another; (d) All this is due to internal essences that make the members of each category what they are. When our genome or DNA are perceived to be these essences and when this kind of thinking is applied to social categories such as race and ethnicity, a view that we call “sociogenetic essentialism”, it can be highly problematic as it can form the basis for discrimination and exclusion. We argue that the use and reference to ancestry informative markers, unless clearly explained, may be misinterpreted due to a sociogenetic essentialist bias as confirming the genetic basis of social groups.

## Introduction

Advances in DNA testing technologies have made ancestry testing a popular tool for exploring one’s past. These tests provide insights mostly about geographic ancestry, but can have implications for understanding race, ethnicity, and identity. However, the way individuals interpret these results is shaped not only by the underlying science, but also by their own psychological frameworks. One such framework, actually a cognitive bias, is (psychological) *essentialism:* the belief that some categories are underpinned by an immutable and inherent essence. This paper explores the interplay between psychological essentialism and DNA ancestry testing, raising critical questions about how the results of ancestry tests could influence perceptions of group identity and belonging. We begin by describing psychological essentialism and its relevance to the perception of social categories. We then examine the perceived nature of social group "essences" and discuss how ancestry informative markers (AIMs) may be misinterpreted as such, given their use as genetic proxies for race or ethnicity. Finally, we analyze how individuals interpret ancestry testing results, highlighting the ways in which psychological essentialism can shape both understanding and misinterpretation.

This discussion sheds light on the broader social implications of genetic ancestry testing in reinforcing or challenging essentialist views of identity. Overall, we argue that psychological essentialism may influence how individuals perceive and interpret the results of DNA ancestry testing. The belief that certain social categories, such as race and ethnicity, have inherent and immutable "essences"—can shape the way people make sense of genetic information, often reinforcing ideas that social categories may have a genetic basis, a view that we call “sociogenetic essentialism”—a sub-type of psychological essentialism. We contend that while DNA ancestry tests provide probabilistic insights into geographic ancestry, the language of the reported results may reinforce interpretations of these results and the related conclusions through an essentialist lens, attributing to them greater determinative power over personal identity and group membership than is scientifically warranted.

Essentialism is a key topic both in history and philosophy of science, as well as in cognitive psychology, and it is important to clarify what one means when using the term, and in which historical context it is being considered (see for instance, Kampourakis, 2015a, 2015b; Ware et al., 2015; Shtulman, 2015). Therefore, it is necessary to clarify some interrelated concepts that are sometimes used interchangeably: psychological essentialism, social essentialism and genetic essentialism. Whereas all three describe how individuals perceive categories, particularly human traits or groups, as having an inherent and immutable essence, they are also distinct:

- Psychological essentialism, which encompasses social and genetic essentialism, is the belief that certain categories have an underlying, fixed essence that defines their true nature. When people think in essentialist terms, they believe that members of a category share some intrinsic properties that determine their identity and characteristics, regardless of external factors or individual variation, as in the belief that all members of a species have the same basic traits.
- Social essentialism is psychological essentialism applied to specific socially constructed categories, such as race, ethnicity, gender, or class. It involves the belief that social groups have inherent, essential characteristics that define the identity, traits, and behaviors of their members. Through the lens of social essentialism, socially constructed categories are perceived, not only as real/natural, but also as grounded in a deep and immutable essence. Such examples are the beliefs that African Americans are naturally more athletic or that Asians are inherently better at mathematics.
- Genetic essentialism is the belief that an individual’s identity, abilities, and traits are largely determined by genetic factors. This view often leads people to see genes, genomes or DNA as the essences and therefore as the primary or sole determinant of specific features such as intelligence, educational attainment, behavior or disease. In these cases, genes, genomes or DNA serve as the placeholders of our internal essences.

**Fig. 1:**
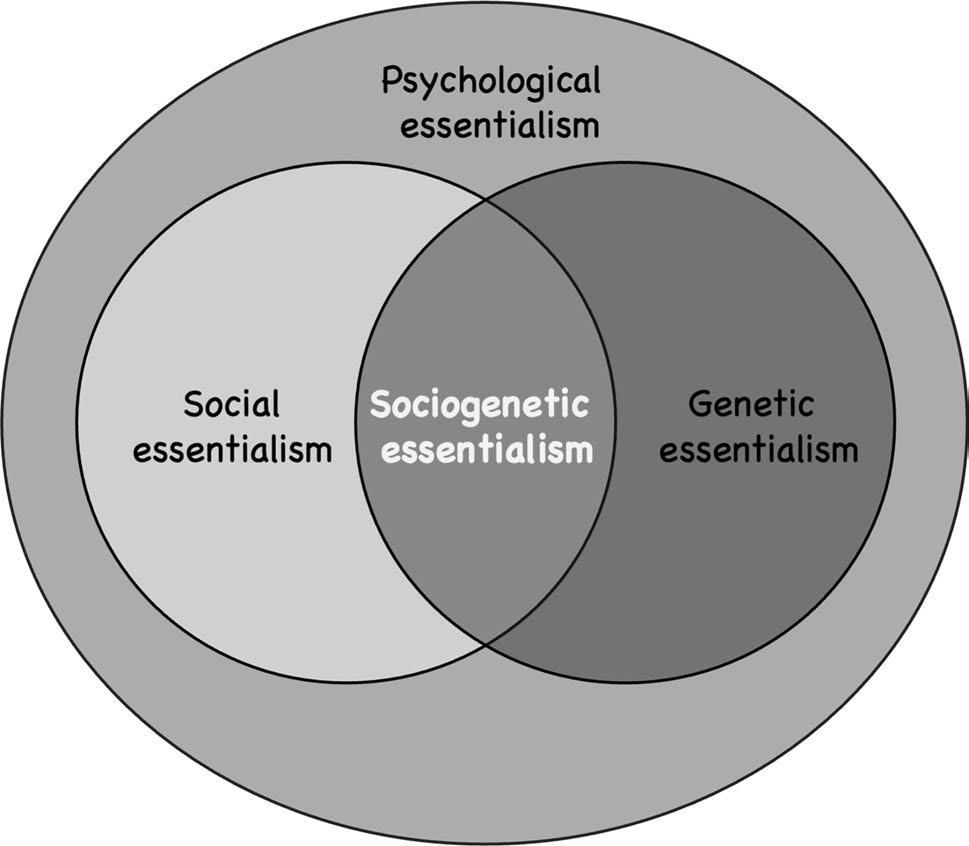
Sociogenetic essentialism, as a subset of psychological essentialism, at the intersection of social and genetic essentialism

As mentioned, all three types of essentialism are based on the underlying assumption that categories have an inherent and fixed essence, which leads to the belief that group differences are natural, immutable, and biologically or intrinsically determined. Psychological essentialism is the broadest view and refers to the general cognitive tendency to think that categories (social or otherwise) have a fixed essence. Social essentialism and genetic essentialism are more specific. On the one hand, social essentialism specifically focuses on the social categories that people are part of, such as race, gender, or ethnicity, and how they are perceived as having immutable characteristics. There are several internal features that can serve as the placeholders of essences; for example, references to “blood”. On the other hand, genetic essentialism narrows the essentialist belief to genetics, asserting that genes, genomes or DNA determine group membership and individual characteristics. What we focus on, in the present paper, is the intersection of social and genetic essentialism: the specific case of genetic essentialism of social groups—when membership in a social group, such as race or ethnicity, is perceived to stem from a genetic essence, such as genes, genomes or DNA, which we call *sociogenetic essentialism* (Fig. 1). A potential implication of sociogenetic essentialist thinking is the conclusion that DNA ancestry tests can definitively reveal someone’s racial or ethnic identity.

Equipped with the lens of sociogenetic essentialism, the present paper bridges psychological studies with studies of genetic ancestry testing, offering a nuanced examination of how essentialist thinking interacts with the interpretation of genetic data. It synthesizes insights from psychology, sociology, and genetics to illuminate the ways social perceptions of race and ethnicity are constructed and influenced by scientific advancements, emphasizing the importance of understanding psychological factors that shape how genetic information is interpreted. By highlighting the potential for DNA tests to be interpreted in essentialist terms, the paper underscores the need for critical engagement with how genetic technologies are marketed and discussed in public discourse.

Whereas scientists are largely in agreement that any attempt to divide humans into biologically distinct and somewhat stable groups is scientifically unwarranted (Kampourakis, 2023), this still seems to be counterintuitive for lay people. The scientific stance on the constructed nature of human social groups is much harder for the general population to accept compared to the idea that certain human sub-groups are real, or natural, in the sense that membership identity is heritable and stable over time. Furthermore, people believe that such social identities can be scientifically detected, even if the means to do so have not yet been sufficiently developed. The widespread nature of these beliefs and their emergence at a young age motivated scholars to study them as intuitive forms of reasoning, mostly under the umbrella of the study of Psychological Essentialism (Medin & Ortony, 1989, pp.184–185).

## What is psychological essentialism?

Essentialism is the idea that entities have essences, i.e., underlying properties which are characteristic of them (Wilkins, 2013). Psychological essentialism is the idea that humans believe that essences exist, and that the essences of organisms are fixed and unchanging, and as such they characterize organisms despite any superficial changes they may undergo (Gelman, 2004). People often categorize other people almost at first sight and they also, effortlessly, generalize from very little interaction or few observations to the rest of the constructed category members. For instance, clothing stores and toy stores are (non-scientific) evidence for the existence of commonly accepted expectations as to what boys would, and maybe should, prefer to wear and play with, as opposed to girls’ preferences. Up to this point in time, most people have not abandoned the use of such categories as gender for making inferences about individuals, beyond the scientifically accepted inferences resulting from sex differences. Thus a baby with pink clothes will be generally supposed to be a girl rather than a boy that, for whatever reasons, happens to be wearing pink clothes. Other social categories are also treated in this way, although not necessarily always to the same degree. Considering religious groups, occupational categories, races, nationalities, and ethnicities, to name a few, these social categories differ in the level to which people consider them as natural kinds (heritable, immutable) and cohesive (homogenous), and overall biological by essence. To treat or consider a social category as highly natural and cohesive, is to essentialize it, or in other words, to engage in Social Essentialism (Haslam et al., 2000).

It is important to briefly explain here why this phenomenon of perceiving a category as a natural kind is referred to as Essentialism. The idea is that humans do not only consider the group as cohesive and natural, they also assume that each member of the group contains an essence, even if we do not know what it is or where to find it, which gives rise to the shared features and the group identity. One prominent theory about how humans represent the essence is called Placeholder theory (Medin & Ortony, 1989; explained in depth later in this paper), and what is important to know about it for now is that this placeholder can be replaced or filled with different ideas- at times people could believe that the essence is in the blood, maybe the brain or the heart, and nowadays, a common filler for the placeholder is DNA. We get back to this idea after discussing the naturalness and cohesiveness of categories.

What does it mean to treat a social category as natural and as cohesive (i.e. essentialize it)? In the first systematic study of social essentialism, Haslam et al. (2000) studied the extent to which humans essentialized 40 different social categories (e.g. females, introverts, Catholics) that represented 20 social domains (e.g. genders, personality, religions). They created an Essentialist Belief Scale (EBS) of nine elements, which they based on a review of the existing literature on Essentialism from psychology, philosophy, and social sciences. Prior to their work, there was a long history of research and theory about Essentialism, but there was also a lot of variability in the ways it was defined across these disciplines, leading Haslam and his colleagues to attempt to unify the language and the theories into an overall measure of Essentialism. The 9 elements which encompassed most of the previous work made up their full EBS. This scale is still widely used by researchers, either in its entirety or in modified versions, to evaluate Essentialist tendencies and to correlate those with other beliefs and attitudes about social groups, as we will demonstrate later in this paper.

The nine Essentialism elements are:

1. *Discreteness*—where a social category falls on a scale between having sharp boundaries, so that you are either in or out, and being rather fuzzy, where membership has a degree to it.
2. *Uniformity*—how similar members of a social category are to one another, or whether there is a large degree of diversity.
3. *Informativeness*—how many judgements one could make based on a person’s group membership.
4. *Naturalness*—where a social category falls on a scale between ‘natural’ and ‘artificial’.
5. *Immutability*—how easy it is to leave the group and become a non-member.
6. *Stability*—whether a social category has always existed pretty much as it is, or whether there was a time when it did not exist or its characteristics were significantly different.
7. *Inherence*—whether there is an underlying reality that is shared by group members, even if they have both similarities and differences on the surface.
8. *Necessity*—whether there are any particular characteristics that group members must have in order to be members.
9. *Exclusivity*—whether the category prohibits members from belonging to other categories or does it allow members to belong to other categories.

In order to create a broad list of forty social categories that would represent the wide range of social domains, Haslam and his colleagues asked ten students to come up with two categories for each of the 20 domains and they selected the two most common categories of each domain to be part of their survey. Their final list included the following 40 social categories (within parentheses) organized into the 20 social domains (in bold) they belong to: **age groups** (`old people’, `young people’), **dietary groups** (`meat-eaters’, `vegetarians’), **ethnic groups** (`Asians’, `Hispanics’), **gender groups** (`males’, `females’), **intelligence groups** (`people of average intelligence’, `smart people’), **interest groups** (`movie buffs’, `sports fans’), **language groups** (`English-speakers’, `Spanish-speakers’), **disabilities** (`blind people’, `paraplegics’), **diseases** (`AIDS patients’, `cancer patients’), **occupations** (`blue-collar workers’, `doctors’), **personality** (`extroverts’, `introverts’), **physical appearance** (`attractive people’, `ugly people’), **physiques** (`large people’, `small people’), **political groups** (`liberals’, `Republicans’), **psychiatric disorders** (`depressives’, `schizophrenics’), **races** (`Black people’, `White people’), **regions** (`Easterners’, `Mid-westerners’), **religions** (`Catholics’, `Jews’), **sexual orientations** (`heterosexuals’, `homosexuals’), and **social classes** (`lower-class people’, `middle-class people’).

In their first use of the EBS, Haslam and his colleagues (2000) asked forty students to evaluate the above mentioned 40 social categories (each participant saw only half of the categories, one of the two categories in each of the twenty domains) based on the nine elements of the EBS: *Discreteness, Uniformity, Informativeness, Naturalness, Immutability, Stability, Inherence, Necessity*, and *Exclusivity*. While the forty social categories varied widely in their scores on the nine elements, here we focus on another aspect of the findings. Haslam and his colleagues found significant correlations between some of the nine elements, but not between all of them. Their principal component analysis revealed two distinct dimensions (clusters), each with high internal correlation among the elements. One dimension included discreteness, naturalness, immutability, stability and necessity, which were taken to represent the extent to which a social category was perceived similarly to a natural kind—having sharp boundaries and clear necessary features, as well as being hard to change. The second dimension included uniformity, informativeness, inherence and exclusivity, which were taken together to signify the level of cohesiveness (coherent entity) of a social category—how similar members are, so that inferences can be made from one member to another, an exclusive similarity that is the result of a inheritable underlying causal reality.

Out of the 40 social categories, the two that are most relevant to this paper are race and ethnicity, as they are implied by the way the companies present their ancestry results – although we must note that recently there is an increasing tendency to use geographical descriptors (for instance, Ancestry now refers to geographic origins rather than ethnic origins). The results from Haslam et al. (2000) suggest that race and ethnicity are perceived relatively high on the natural kind dimension. This means that people generally think that race and ethnicity are natural (rather than constructed or artificial) categories, with sharp boundaries between the various groups/races/ethnicities and shared necessary features among the members of each group, so that it is a clear cut which race/ethnicity one does or does not belong to. These social categories are seen as unchangeable during one’s lifetime and as if they have always existed in the world in a stable form throughout human history.

We were struck by the parallels between this pervasive naturalistic perception of race and ethnicity and people’s expectations of and conclusions from their DNA-based ancestry reports. These reports may be perceived and interpreted as revealing a person’s ethnic or racial identity, on the basis of DNA data. Because DNA is something inside us, it is a common filler for the intuitively perceived essence-placeholder, believed to hold the key to group identity. Therefore, it might be intuitive to interpret an ancestry DNA results’ report in an essentialist manner, both confirming our existing ideas and possibly strengthening those essentialist perceptions. This entails that the ethnic or racial groups to which a person is assigned may, in the end, be perceived, among other things, as natural, discrete, and internally homogeneous. It is therefore interesting to consider research with ancestry DNA test takers in order to explore whether and to what extent they interpret their results in an essentialist manner. But before we do so, it is useful to understand what Social Essentialism is and what it entails. Next, we provide further scientific evidence for the naturalistic essentialist way in which humans perceive racial and ethnic categories, which underlies the tensions between constructed and naturalistic views of race and ethnicity.

## Are social categories perceived as constructed or as natural?

Since the 2000s, the study of social essentialism flourished and developed into various kinds of social categories such as race, gender, religion, socio-economic status, and sexual orientation, to name a few. The Essentialist Belief Scale (EBS), which is described in details above (Haslam et al., 2000) has featured prominently in many of these studies, either in its entirety or in modified versions. Other widely used methods among scholars of social essentialism have included the Switched at Birth task (SAB) and the Race Conception Scale (RCS), which we will describe in this section. The focus of this section is on studies that give us a clue about the ways in which people perceive ethnic and national groups. More specifically, we are interested in whether these categories are essentialized and if so, in what ways. The reason for focusing on these social categories lies in the ways that ancestry testing companies present their results to test-takers, as we describe later in this paper.

One of the early studies of Psychological Essentialism of ethnic groups, performed by GilWhite in 2001, employed the Switched at Birth (SAB) task and presented participants with a juxtaposition of ethnic Mongols and ethnic Kazachs in Mongolia. In the SAB paradigm, participants usually read a short story about two couples who come to the same hospital to give birth. Unbeknownst to them, their babies are switched and each couple takes home the baby of the other couple and raises them as their own. At this point the participant is asked to speculate about the properties of the child on a range of domains. For example, if the birth parents are outgoing and extroverted and the adoptive parents are shy and introverted, the participants need to decide what type of personality the child is more likely to have between that of the birth parents and that of the adoptive parents. Alternatively, if the birth parents like soccer and the adoptive parents like basketball, the participants are asked to determine whether the child will like soccer or basketball. According to the level that a participant attributes birth parents’ properties to the child, they are considered to hold higher or lower essentialist views about humans. Researchers have the flexibility to choose which properties they ask about and which domains of social categories they invoke. In the case of GilWhite’s (2001) study, in addition to the SAB question, he asked ethnic Torguud (another ethnic group in Mongolia) participants what is the ethnicity of a child that was born to a Kazach father and a Mongol mother. Based on the answers of a hundred participants across two studies, he learned that in Mongolia, ethnicity affiliation is patrilineal, i.e. it is determined by the father’s ethnic affiliation. Even when participants were told that a child of a Kazach father and a Mongol mother is raised only around Mongols, has never seen a Kazakh, and learned Mongol customs and language, participants still attributed a Kazakh ethnicity (descent based—patrilineal) to the child.

Finally, the SAB version in GilWhite’s study presented participants with a scenario in which a Kazakh couple has a young child (less than one year old) they do not want to keep and give it away for adoption. The adoptive parents are not Kazakh, but Mongol (as a control, GilWhite also presented the opposite scenario, where birth parents were Mongol and adoptive parents were Kazakh, and he also changed the child between male and female). As in most SAB paradigms, the child is unaware of the adoption and believes their mother, and, in this case more importantly, their father to be Mongols. The child never meets any Kazakhs and, in this scenario, GilWhite did not ask participants to speculate about the language and customs that the child will learn. Instead, GilWhite provided the information that the child will learn their adoptive parents’ customs and language, and asked participants what is the ethnicity of the child. Participants maintained the idea that ethnicity was based on biological descent by showing a preference to the birth-parents’ ethnicity, even though that ethnic background was unknown to the child at question, and furthermore, even though the child did not display physiological and behavioral phenotypes uniquely consistent with the assumed biologically-based ethnicity. GilWhite concluded that Torguuds treated ethnicities as natural kinds, similarly to the way they perceive species, and therefore they also tend to essentialize those ethnicities.

It should be noted that the concepts of race and ethnicity are often confused or used interchangeably. On top of that, in different parts of the world, these terms are used to different degrees, but often refer to similar delineations between groups of people. A lot of the work on social essentialism has been produced by academics based in the USA, where ‘race’ has been the more common social domain to study within social essentialism. We therefore discuss another scale that was developed to measure social essentialism on ethnicity/race—the race conception scale (RCS). Williams and Eberhardt (2008) developed and validated a scale to measure the extent to which an individual would see race as biologically based. The 22 scale items overall capture how biological, natural, easily discernible and stable race is perceived to be. Examples of items are “It’s impossible to determine how a person will be racially categorized by examining their DNA.” and “A person’s race is fixed at birth.”, and they are rated on a 7-point scale (from 1 = strongly disagree to 7 = strongly agree, with some that are reverse coded).

People’s ideas about natural divides between human populations, especially along ethnic boundaries, can be so compelling that it even biases perceptions of such relatively-recent socially constructed categories like citizenship. Devos and Banaji (2005) studied the association of different ethnic groups (Asian, Black, and White) with the category “American” using both implicit and explicit measures. A group of 135 students were asked to rank each ethnicity on a 7-point scale (from 1 = Not at all American to 7 = Absolutely American). Even though participants were asked to consider individuals who are citizens of the United States, as well as who were born and lived in the United States, their responses indicated a significant hierarchy, wherein White Americans were considered more “American” than Black Americans, who in turn were considered more “American” than Asian Americans. Devos and Banaji (2005) showed that even though participants did tend to claim that all ethnicities should be treated equally, they still had an idea they are somehow different- “not in rights and liberties but in the degree to which they embody the concept “American.””.

This taps into the idea that people do think that there is a prototypical form of relatively modern nationalities, and that part of it is ethnic/racial. And, interestingly, this came out in a quite “explicit” measure, reported in the same publication (Devos & Banaji, 2005). Participants were asked to “bring to mind individuals who were born in and are citizens of the United States. “In your mind, how “American” are people who belong to the following groups? That is, how strongly are they identified with America and all things American?” Each participant answered this question three times—once for each of the categories: African, Asian, and White American. These findings imply that even if, for example, scientists claim that a social category, for instance “Swedish”, is completely socially constructed, and they expect people to accept it as such (i.e. solely based on citizenship), the general population is more likely to have an idea of a prototypical “Swedish” person. Similar results were found when Devos and Banaji (2005) asked about the three ethnic groups’ association with American culture, with Asian Americans being less associated with “American culture” than either White or Black Americans. The difference between the ethnicities was also present in implicit measures such as the Implicit Association Test (IAT), with White Americans being more associated with the concept “American” than either Black or Asian Americans.

Members of a minority ethnicity are often faced with a denial or a suspicion about their membership in the constructed in-group, so much so that Cheryan and Monin (2005) have coined the term “identity denial” to describe this acceptance threat. A common question posed to Asian-Americans is “where are you really from?”, revealing the questioner’s intuition that someone who’s appearance does not match that of the in-group prototypical American (in this case – White/European) is not a real member/American.

In their study, Cheryan and Monin (2005) asked both White-American and Asian-American students to rate 8 faces, for which they received made-up names and places of birth (in or out the US), on a 7-point scale, for five attributes: attractive, intelligent, happy, American, and conscientious. The question of highest interest was how ‘American’ they judged these eight individuals to be. Participants saw White people who were labeled as born in the US and some in the UK, Asian people who were labeled as born either in the US or Taiwan, and they also saw Black and Hispanic faces who were labeled as born in the US. Similarly to the results from Devos and Banaji (2005), Cheryan and Monin (2005) found a hierarchy where White faces, who were labeled as US-born, were rated as most American, Asian faces of American-born identities were rated significantly less ‘American’ than their White counterparts, and Black and Hispanic faces were in the middle—significantly less ‘American’ than White faces, but significantly more ‘American’ than Asians. These patterns were observed both in results from White participants and Asian participants. Another part of their study evaluated the extent to which members of different ethnic groups are misrepresented by others as being from another country (as opposed to being from the US) and/or as being a non-native English speaker. Cheryan and Monin (2005) asked students to recall first meetings with strangers and choose all the ways in which they were misperceived by those strangers out of a list of 17 options plus the option—‘other’. The two ways of being misperceived that were of most interest were, as explained above, whether people were assumed to be non-native English speakers and/or to be from another country. Asian students reported almost 5 times more often being misrepresented as coming from another country, and almost 10 times more often as being non-native English speakers.

All in all, as we have demonstrated above, people have strong intuitions about how different ethnicities/races fit into nationality categories, in a way that takes priority over mere citizenship laws.

## What is perceived to be the essence of a social group?

Social essentialism, as already stated, is a special case of the broader domain of Psychological Essentialism (Medin & Ortony, 1989; Gelman 2003)—the study of the psychological representation of categories, regardless of whether they are consistent with metaphysical categorizations. People create categories almost effortlessly, and scientists are set to understand the psychological principles by which people delineate those categories. A salient idea in many studies of psychological essentialism, the Placeholder, was coined by Medin and Ortony (1989), and proposed that a part of the knowledge about a concept is an essence-placeholder (that can be filled by different beliefs), which is the part that people will use to determine category membership of anything. However, different kinds of “things” may be categorized differently, based on the belief that fills in the essence-placeholder. For example, human-made artifacts (hammers, phones, cars) are often categorized by their function or purpose, and in that case we would say that people fill in the essence-placeholder with the belief about the purpose of an object. On the other hand, in the case of natural objects, like gold and coal, the placeholder might be filled with beliefs about the materials that the objects are made of.

The theory of an essence-placeholder implies that even if people cannot articulate what it is that makes a social category homogenous and distinct from another social category, they would nonetheless assume that there is an essence which is the real cause for that distinction. This structure of knowledge, the essence-placeholder, allows for the belief that someone else, like an expert or a scientist, could potentially find out what the essence really is. Therefore, non-experts do not need to know exactly how that essence is causally related to the external features they use for categorization. For example, if doctors or scientists discuss blood as a source of illness, people might use that scientific information to fill in the essence-placeholder, especially if it fits their existing intuitive assumptions about what features should be part of the essence. Sousa et al. (2002) summarized the schematic assumptions that were associated with the idea that the essence has a causal effect as:

1. The natural kind identity of a baby is caused by the fact that a natural kind X gave birth to it, because the baby inherits X’s essence.
2. A natural kind X grows to be an adult X, because its essence gives the innate potential to be so.
3. In normal circumstances, the essence of natural kind X causes X to have characteristic A.

When scholars in the natural sciences discuss a feature of an organism’s body in terms of it being inheritable from one generation to the next, and related causally to some other features like diseases, these ideas “hijack” humans’ existing psychology and their predisposition to fill in their essence-placeholder, with the result being that those ideas are easily endorsed, even if the people who endorse them do not necessarily properly understand the scientific idea and cannot articulate the full pathway by which it causes the external features. In other words, people categorize based on certain features, they assume an essence that is the cause for the structure of their categorization, and they post-hoc incorporate scientific ideas to explain or justify their categorization as a natural state of the world, rather than a constructed structure that they enforced on reality.

Findings from a community in Brazil support this idea of relying on experts. Sousa et al. (2002) tested children (4–7 year olds) and adults using the Adoption task (similar to the SAB task). They presented participants with a scenario in which a cow gave birth to a baby and died immediately after birth. The baby was “found and taken right away to live with pigs in a place where there are lots of pigs”. The participants were shown a drawing of the cow that gave birth to the baby, a drawing of the pig that took care of the baby, and they were told that the baby never saw another cow for the whole time it was growing up. After hearing the story and answering comprehension questions correctly, participants were asked to determine whether the baby would develop traits and behaviors of the birth-parent (the cow) or the adoptive-parent (the pig). Across all four behaviors and traits, all participant groups were more likely to choose similarity to the birth parents over the adoptive parents. These four behaviors and traits included both previously known traits (straight vs. curly tail) and behaviors (produce a moo or an oink sound) as well as previously unknown (made up) traits (whether the baby’s heart will get flatter or rounder when it sleeps) and behaviors (whether the baby will chase chickens or ducks).

In the last part of their survey Sousa et.al. (2002) asked participants two kindhood questions. First, they were asked to point to the drawing (pig or cow) to indicate what kind of animal the baby is once it is all grown up. Secondly, they were told another follow-up story about a time during the baby’s development when it got sick and a doctor came and took out all its “old” blood, which it got from its birth mother, the cow, when it was born, and replaced it with all “new” blood from the adoptive mother, the pig. This story was followed by another kindhood question, requiring the participants to point to the drawing of the kind of animal the baby will grow up to be. Before hearing the story about the blood, all age groups were significantly more likely to claim that the baby will grow up to be a cow, but after hearing about the blood extraction and transfusion, only the adult group maintained that idea, while all children groups were more likely to choose the identity that corresponds with the kind of blood the baby has—after replacing its cow blood with pig’s blood, the children in the study claimed the baby will grow to be of the kind of pig. As Sousa and his colleagues (2002) explain, children in these societies, and in many other societies, hear the adults talk about shared blood as an explanation for family ties, and they might take it more literally than the adults around them do. For those children, who have an essence-placeholder to fill, the idea of blood, in the way it is communicated around them, is an excellent candidate to fit right into that void.

A later study (Waxman et al., 2007) used the same Adoption paradigm to generalize the findings about the blood as a filler for the essence-placeholder to other communities. In one study, they compared rural Native Americans (Menominee) with rural European Americans. While the adults in both communities rejected the idea that blood transfusion changes an animal’s kind, they did find a cultural difference between children from the different communities. Menominee children were more likely to accept a “kind” change following a blood transfusion than their European-descent neighbors. This difference in the likelihood of children to treat the blood as the filler of the essence-placeholder, corresponds to the fact that the Menominee community determines tribal affiliation based on Blood Quantum Measures—the proportion of documented ancestors who are full-blood Native Americans. These findings are similar to those from the Brazilian children (Sousa et al., 2002), but they add the idea that the essence-placeholder filler can vary based on commonly acceptable ideas in a community.

As we explained above, the combination between an essence-placeholder and scientific knowledge about a feature that is inheritable, stable throughout development, correlated to external features, and possibly hard to observe has the potential to create a strong validating belief about the naturalness of categories. We have discussed the idea of blood above, but there are other candidates like the heart, the brain, the liver etc. One very successfully endorsed candidate is DNA. Studies have shown that even young children in Westernized societies are familiar with the idea of DNA or genes, even if they do not have a very scientifically rigorous understanding of its structure and function. The concept of DNA “checks all the boxes” for an ideal candidate to fill in the essence-placeholder—it is largely unobservable, heritable, and discussed in relation to various external features. It should be of no wonder that many people hold the belief that DNA can confirm their already existing beliefs about the organization of the world into natural categories. This idea has been described as genetic essentialism.

There is a lot of research that has shown that essentialism has a strong impact on people’s understanding of heredity. Genes are thus often perceived as the essence of being and the source of whatever we are, think, or do, to the extent that it is thus possible to infer one’s characters and behaviors from one’s genetic makeup. In particular, it has been suggested that genetic essentialism may make people think of genetically influenced traits as unchangeable and prescribed, consider the relevant genes as being the fundamental cause of the respective characteristic, view groups that share an inherited characteristic as being homogeneous and discrete from other groups, and perceive characteristics as more natural if they are genetic (see Heine, 2017; Heine et al., 2019 for overviews).

As already explained, psychological essentialism and genetic essentialism are closely related, with the latter being a specific manifestation of the former in the context of genetics. Psychological essentialism refers to the cognitive tendency to believe that categories have an inherent, unchanging "essence" that defines their identity and properties, often assumed to be biologically grounded and causally responsible for observable traits. Genetic essentialism narrows this down, attributing such "essence" specifically to genes, genomes or DNA and promoting the belief that genetic information is the primary determinant of an individual’s identity, abilities, behaviors, and membership. Both frameworks thus contribute to the perception of some categories as natural, discrete, and biologically rooted. The key difference is that psychological essentialism, as well as social essentialism, is referentially indefinite, that is, it does not specify what the presumed essences are. In contrast, genetic essentialism is referentially definite by considering genes, genomes or DNA as these essences.

Let us consider a few studies on genetic essentialism (Dar-Nimrod et al., 2014). Three studies were conducted in order to investigate what implications people perceive from a supposed genetic etiology for obesity. In the first study, 131 undergraduates indicated whether obesity originates from a genetic disposition or environmental causes, as well as whether or not they believed that obese people can control their weight. The results suggested an association between a belief in genetic etiology for obesity and a belief that obese people cannot control their weight. These associations were further investigated in a second study, in which 143 undergraduates were asked to express their beliefs about an obesity-related phenomenon (metabolic rate) in the light of particular explanations. The results indicated that a genetic attribution for high metabolic rate was interpreted as more important than an experiential attribution. Finally, in a third study, 162 undergraduates read one of three fictional media reports presenting a genetic explanation, a psychosocial explanation, and no explanation for obesity. The results indicated that participants who read the genetic explanation ate significantly more on a follow-up task.

One major issue is that such messages can be found in textbooks. For instance, a study with 135 7th-9th graders in the USA found a significant effect of racial-oriented biology texts on the perception of different behaviors and competencies (such as intelligence) between races based on genetic variations, concluding that biology education can enhance views about racial inequality (Donovan, 2016). Indeed, another study with 43 American 8th graders, who were taught either through a racialized or a non-racialized textbook on human genetic diseases, concluded that biology textbooks may strengthen essentialist conceptions of races (Donovan, 2014). Recent research by Brian Donovan has shown that such attitudes can be moderated if students are taught about human genomic variation and that most of it is shared among humans, independently of race or continental groups (Donovan et al., 2019, 2020, 2021).

But what exactly might people perceive as constituting this essence with respect to social categories such as race/ethnicity? Ancestry informative markers are a possible candidate.

## Ancestry informative markers as potential perceived genetic essences of race/ethnicity

Ancestry-informative markers (AIMs) are sets of DNA polymorphisms that are found in substantially different frequencies in populations from different geographical regions of the world. AIMs are thus assumed to be useful for estimating the geographical origins of the ancestors of an individual typically by continent of origin (Africa, Asia, or Europe). Table 1 presents a recently published set of AIMs consisting of 12 SNPs (single-nucleotide polymorphisms), based on data from the 1000 Genomes Project (1KGP), which according to the researchers: “… gives a perfect continental classification on the 1000 genomes dataset (if admixed Americans are excluded).” Why were admixed Americans, who in that particular sample were referred to as individuals coming from Mexico, Puerto Rico, Colombia and Peru, excluded? Because during the various trials that the researchers did, including these groups made it difficult to find AIMs which could distinguish the continental origin of ‘Americans’ (with small error), but excluding these admixtured groups allowed them to get “cleaner” results. The explanation they provided was that Americans had a mixed background compared to the other groups, and so distinction was not possible (Pfaffelhuber et al., 2020).

**Table 1:** A set of 12 SNPs, and their frequencies within each geographical population, that can serve as ancestry informative markers (AIMS) for four continental regions: Africa, East Asia, Europe, South East Asia. The authors of the study cited here argued that these 12 SNP's were sufficient for correctly classifying all African, European, East-Asian, and South-Asian individuals in the 1000 Genomes project (Data reprinted with permission from Table 3 of Pfaffelhuber, P., Grundner-Culemann, F., Lipphardt, V., & Baumdicker, F. (2020). How to choose sets of ancestry informative markers: A supervised feature selection approach. *Forensic Science International: Genetics*, *46*, 102,259)

SNP	Africa	East Asia	Europe	South East Asia
rs2814778	0.964	0	0.006	0
rs868179	0.736	0.01	0.098	0.329
rs16891982	0.036	0.006	0.938	0.059
rs6899550	0.944	0.029	0.292	0.421
rs73591062	0.293	0.002	0.005	0.275
rs59700332	0.778	0.002	0.038	0.044
rs1426654	0.926	0.988	0.003	0.315
rs376893831	0	0.001	0.0	0.224
rs368844055	0	0.002	0.001	0.539
rs201922096	0.905	0.924	0.11	0.635
rs74521229	0.008	0.002	0.195	0.442
rs6053171	0.393	0.982	0.083	0.598

Table 1 presents frequencies (from the 1KGP data) of the reference alleles in the four continental regions. Here is an example of how it works: rs2814778 is highly likely to be an African ancestry marker since the reference allele almost only appears there; rs1426654 is capable of separating (to some degree) African and East Asian ancestry from the other two, while rs73591062 has fairly low power to distinguish African and South East Asian ancestry from the other two. In combination, based on which AIMs they are found to have, individuals can be probabilistically assigned to the four continental regions. It is important to note that assignments based on a single marker are hard to achieve, and that one has to consider all the AIMs together (Peter Pfaffelhuber, personal communication).

The first obvious application of AIMs is inferring continental ancestry. In one study, the researchers were interested in how small an AIM panel of SNPs could be. They suggested that the selection of AIMs should focus on those SNPs with the largest allele frequency differences between the continental regions of interest. This was necessary in order to achieve the desired resolution, that is, be able to clearly distinguish among the various regions. They noted that “Such high resolution is required because genetic diversity of human populations follows gradients or geographic clines within and among continents rather than specific clusters or clades.” A cline is a geographic gradient, in which there is a gradual change in the frequencies of SNPs (or alleles) across geographical space. In such a case, the frequency is high in one region and gradually declines the further away one moves from that region. Clines of genetic variation can be thought of as being analogous to temperature lines on a weather map, which connect places that have the same temperature. Similarly, on a genetic cline there are lines that connect places that have the same SNP (or allele) frequencies. Eventually, the researchers developed a panel of 41 AIMs, which could be used to accurately assign individuals to seven continental regions. But they also noted: “Independent of the statistical method used to determine ancestry and admixture proportions, the results of these analyses depend not only on the informativeness of the genetic markers, but also strongly on the set of reference populations included. An omission of reference subjects from an ancestral group likely leads to misclassification of test subjects with similar ancestries.” (Nievergelt et al., 2013). So, two issues have been raised so far regarding AIMs: the nature of human DNA variation, which is clinal and not clustered, and the representativeness of the samples studied.

Other studies have suggested that it is possible to use AIMs in order to infer ancestry for populations of the same continent. One study used data from Europe. In order to find informative AIMs, the researchers used a set of 70 individuals per population, in order to avoid overrepresenting populations with larger sample sizes, and arrived at a set of 25 most informative markers. However, the researchers noted that “… we treat individual populations as independent, and select markers, which explain equally well the difference between all these populations. This is obviously a problem with closely related populations; we can see from PCA graphs that central European populations are in fact not independent.” Finally, the researchers considered as a strength of their study the fact that the DNA samples they studied had been collected for clinical purposes (anorexia nervosa) and not with the aim to study ancestry. As a result, detailed information about ancestry was not always available. They thus noted: “For these reasons, our data may not be as evenly distributed or as well defined as that used in previous population differentiation studies, in which it is usually required that all four grandparents of the sample are also from the region.” (Huckins et al., 2014). This study thus raised another three important issues: the sample size, the criteria for distinguishing among populations, and the criteria for the inclusion of participants in a study.

Another study came to question the importance of the informativeness of individuals’ AIMs. Its goal was to estimate the number of SNPs required in order to reliably infer genome ancestry using random panels of SNPs (rSNPs) compared to pre-designed AIM panels. Using data from 50 individuals from each of three groups (European, East Asian, and African ancestry), they performed simulation experiments with various combinations of their data. Their conclusion was that panels of 10,000 rSNPs could be used to accurately infer European, East Asian or African ancestry. Even though ancestry inferences were less accurate as they decreased the number of rSNPs used, even panels with only 500 rSNPs produced reliable ancestry estimates, which they compared to those of the predesigned AIM panels. However, the researchers found that below that number, the errors in the ancestry inference increased. They concluded by noting that “Caution should be exercised when inferring ancestry using AIM panels. The concept of ancestry is a complex one and although it can be operational for particular purposes, it can lead to erroneous perceptions of human variability.” (Pardo-Seco et al., 2014). This study therefore showed that AIMs may not be as objective means to identify ancestry as some might think, as random combinations of AIMs may produce seemingly valid estimates.

Perhaps the most important concern about AIMs is ascertainment: the procedure used to find SNPs that vary among different human populations. Molecular evolutionist Robert DeSalle and paleoanthropologist Ian Tattersall (2018; 2022) have argued that AIMs are the outcome of an extreme ascertainment process. This in turn can result in what is described as ascertainment bias: having a dataset that is different from the respective population it is supposed to represent, because the selection of samples resulted in some members of the population being less likely to be included than others. If ascertainment bias affects inferences about the genetics of populations, could the solution be to use whole genome sequencing data? Well, even that might not be enough. In a recent study, the researchers used whole genome sequencing data in order to achieve an unbiased selection of DNA variants. Their conclusion was that the use of predefined sets of SNPs may arrive at an overall accurate representation of the relationships among non-African populations, but not among African ones. The researchers also found that populations in central and southern Africa, the Americas, and Oceania each have tens to hundreds of thousands of “private” DNA variants, that is, variants that have occurred in one lineage but not in others e.g., due to mutations. However, although some of these variants were found at relatively high frequencies, none were found at 100% frequency (when this happens, they are described as “fixed”) in any continent or major geographical regions (Bergström et al., 2020). This simply means that there are no DNA variants that are indicative of a geographical region or population in any absolute sense. AIMs are not biogeographical essences and should not be presented as such.

Eventually, the most important problem with the AIMs is that instead of being understood as replacing race with ancestry, they are prone to being misinterpreted as confirming the existence of races on the basis of ancestry. AIMs are used in order to differentiate among predefined social groups, and the results depend mostly on the scientists’ preconceptions about who belongs to each group. They show us that someone is, say, European or African, Greek or Nigerian, because we selected particular AIMs that we believe reflect those groups. But what the AIMs really reflect are only the choices we made about who should be considered European or African, Greek or Nigerian when we conducted the analysis to select them. The AIMs do not reveal any inherent ethnic identity. Therefore, references to AIMs and biogeographical ancestry can be very misleading, if not understood for what they are. As sociologist Rogers Brubaker nicely put it: “And the parental populations to which autosomal tests assign ancestral proportions may, as noted earlier, be conflated with identically labeled commonsense racial, ethnic, and national categories in such a way that the relative genetic similarity used to construct the former is taken as proof of the biological existence of the latter” (Brubaker, 2015, p.73).

Our concern is that laypeople reading about research using AIMs, not necessary in the original papers but most likely in the media, may misinterpret AIMS as racial or ethnic “essences”. AIMs are genetic markers that provide insights into an individual's ancestral origins, but they cannot fully capture the complexity of race or ethnicity, which is shaped by genetics, culture, history, and personal experiences. AIMs can be indicia for race and ethnicity, in the sense that they can probabilistically indicate to which social group a person belongs. But this entails the danger of misunderstanding them as the criteria, the definitive markers and not just indicators, of race and ethnicity. Such a misunderstanding has the potential to oversimplify the multifaceted nature of human identity and undermines the richness and fluidity of these categories. In other words, the problem with AIMs is that they fit well with our essentialist intuitions, and might be misinterpretated as genetic essences of race or ethnicity, thus overlooking the broader cultural, historical, and political contexts that shape our understanding of race and ethnicity (see Kampourakis, 2023).We think that it is very important to see that researchers are, of course, aware of, and also explicitly discuss in their articles all these issues related to: the clinal nature of DNA variation; the criteria for inclusion in ancestry studies and the assumptions about the ancestry of participants; the sizes of the samples of the studied populations and their representativeness; and how indicative of biogeographical ancestry AIMs really are. Science advances exactly because scientists are aware of the limitations of the methods they use and try to address them. What is of concern, though, is how clear all these limitations and assumptions are to non-experts. As a result, these AIMs might be mistakenly perceived by non-experts as representing ethnic/racial genetic essences, whereas they are really not.

## Psychological essentialism and the interpretation of DNA ancestry testing results

### What do people think that the results of DNA ancestry tests can reveal?

Sociologist Alondra Nelson has investigated a range of reconciliation efforts involving people of African descent, focusing on the intersection of genetic genealogy with cultural and historical reckonings. Among these is a West African religious ritual, or "sara," in which participants who are identified as "DNA Sierra Leoneans" seek to spiritually and psychically connect with ancestors who were forcibly trafficked during the transatlantic slave trade. She has also examined the use of genetic ancestry testing as a legal strategy, where descendants of enslaved Africans attempt to establish genealogical links to argue for reparations for their ancestors’ unpaid labor. Furthermore, Nelson has highlighted more recent efforts in the United States where genetic genealogy has been used to confront the historical reliance of elite institutions on the transatlantic slave trade, fostering initiatives aimed at accountability and restitution (Nelson, 2008, 2016).

In 2015, sociologist Rogers Brubaker pointed out that attention regarding ancestry had already shifted from continental-level categories corresponding to human races, to more specific, smaller-scale categories corresponding to ethnic groups, nations and particular regions. He predicted that this shift was likely to continue with two possible outcomes. On the one hand, it might contribute to naturalizing ethnic and national categories, and even other social categories at lower levels, in the case of people who would be assigned to one such category. On the other hand, it might contribute to undermining notions of pure and discrete racial, national or ethnic categories, in the case of people who would be simultaneously assigned to multiple categories. Brubaker concluded: “No doubt both processes will occur; genetic genealogy takes many forms, and its effects are likely to be contradictory and ambiguous” (Brubaker, 2015, p.74). Well, he could not have been more correct. This is why it is interesting to consider the conclusions of some recent systematic studies about how people interpret their DNA ancestry testing results. Initially, studies focused on people’s perceptions of and attitudes towards the tests. Sociologist Jo Phelan and colleagues (2014) aimed at testing two hypotheses, with respect to admixture tests, which were the focus of the study (The researchers analyzed the responses of 526 people who took part in a survey between April 9 and May 7, 2009). The ‘‘reification hypothesis” suggested that the methods of the tests reify race as a genetic reality by implying that racial groups are fundamentally different so that these differences can be identified by the tests. In contrast, the ‘‘challenge hypothesis’’ proposed that receiving admixture test results may challenge, rather than reify, racial categories, as these results generally report that people have mixed racial backgrounds. An admixture vignette was given to 145 participants, and contained clear and strong statements that represented both hypotheses, such as that ‘‘DNA test measures a person’s racial ancestry’’ representing the reification hypothesis, or that ‘‘mixed ancestry is very common,’’ representing the challenge hypothesis. The effect of the admixture vignette was evaluated in comparison to two other vignettes that represented opposing positions on the relationship between genes and race: the “race as social construction” vignette that was given to 139 participants emphasized broad genetic similarities between racial groups and described race as being socially constructed; and the “race as genetic reality” vignette that was given to 148 participants emphasized broad genetic differences between racial groups and suggested that genomic research confirms the existence of racial groups. There was also a control group of 94 participants to whom no vignette was given. The results clearly supported the “reification” hypothesis. The mean belief in essential racial differences was not significantly different for the participants who read the “admixture” vignette and those who read the “race as genetic reality” vignette. In contrast, the mean belief in essential racial differences was significantly lower among participants who read the “race as social construction” vignette and those who read no vignette at all. The researchers concluded by noting: “… our findings point to the possibility that an unintended consequence of the modern genomic revolution is to magnify the degree, generality, profundity, and essentialness of the racial differences people perceive to exist.” (Phelan et al., 2014).

Another study was conducted by political scientists Jennifer Hochschild and Maya Sen (2015). They focused on African Americans because they considered them as more likely to be interested in DNA ancestry testing than others, as they may not know much about their past because of the transatlantic slave trade. In such cases, DNA tests are perceived as offering the means for finding out more about one’s ancestry. To test the hypothesis that African Americans are more interested in, persuaded by, and influenced by DNA ancestry tests than others, they looked at the 2010 General Social Survey (GSS) module. According to the GSS, 63% of the respondents were “strongly” or “somewhat” favorable to the use of DNA to research one’s ancestry, whereas only 9% were not favorable. However, Black people were more likely (42%) than White people (29%) and Hispanic people (39%) to be “strongly” favorable. In order to achieve a more detailed understanding of how important people thought ancestry test results were for identity, the researchers designed another survey with a random sample of 1095 US adults, consisting of 242 non-Hispanic White people, 201 non-Hispanic Black people, 233 non-Hispanic Asian American people, 205 Hispanic people, and 214 non-Hispanic multiracial people (all of them self-identified). In order to document their views, the researchers developed four vignettes featuring fictitious characters who had just received the results of a DNA ancestry test. Two vignettes (one for assignment to many races and one for assignment to a single race) asked participants to imagine that they were the individual depicted in the vignette, whereas the other two (again, one for assignment to many races and one for assignment to a single race), asked participants to imagine how the person depicted in the vignette would feel after receiving those test results. Overall, the results supported the researchers’ initial hypothesis: 45% of African American people agreed with the statement that the test results would “matter a lot” to identity, compared to 41.5% of Hispanic people, 36.5% of Asian American people, 32.5% of multiracial people, and 24.5% of White people. The researchers concluded that “People want to forge some of the links in the broken chain of their heritage, even if the result is not exactly what they had hoped for.” (Hochschild & Sen, 2015).The above two studies were about the reactions of people to hypothetical situations related to DNA ancestry testing. But, what about actual test-takers?

### How do actual DNA ancestry test-takers interpret their results?

Sociologist Wendy Roth has conducted several studies with people who have taken DNA ancestry tests (2018; 2020). One of those had two parts: an online survey with individuals anywhere in the world who had taken a DNA ancestry test; and a subsequent telephone interview with some of them (The online survey was accessible between April 2009 and March 2011). Overall, 482 DNA ancestry test-takers, most of them living in the USA, completed the survey, which included several open-ended questions about the participants’ racial and ethnic identities before taking the test, how these changed after receiving the results of the test, and how these results affected their sense of identity, their activities and their friendships. Before taking any genetic ancestry tests, these people identified as follows: 70.5% as White only; 6.4% as Black only; 1.9% as Asian only. In addition, 8.7% identified as Hispanic/Latino who might be of any race, 5.4% identified with more than one of these groups, and 3.3% identified as White and Native American (whereas only 0.8% identified as Native American only). Interestingly, 57.5% of these people mentioned their curiosity about their racial or ethnic ancestry as the reason for taking the test; and only 8.7% of participants said that they took the test in order to claim membership in a particular racial or ethnic group. The results? Overall, 61% of participants reported that their ethnic or racial identities did not change after testing; in contrast, 19.3% reported that they had thought of their race differently after the test, 34.7% said the same for ethnicity and 14.9% said this for both. The only group that differed significantly from this average were those who had initially identified as Native American only, with three quarters of them experiencing an identity change both for race and for ethnicity (Roth & Lyon, 2018). We should note that Roth and colleagues considered the division into races as being related to biological characteristics, whereas the division into ethnicities as being related to shared ancestry, history and culture.

Subsequently, 100 people from the USA participated in two rounds of telephone interviews. They identified as follows: 41 as mixed race, 33 as White only, 18 as Black only, 3 as Hispanic/Latino only, 5 as Asian only.^1^^]^ The first interview focused on participants’ ethnic and racial identity change throughout their lives. Overall, 36% of participants underwent an identity change due to their test results, with 22% of participants experiencing a racial identity change and 16% an ethnic identity change. In contrast, 7% of participants considered their results to confirm their pre-existing identity. Among those who had identified as White only before testing, 51.6% experienced an overall identity change, whereas among those who had identified as Black only before testing, 16.7% experienced an overall identity change; all the other groups fell somewhere in between. In general, participants experienced an identity change with respect to race slightly more than ethnicity (22% and 16%, respectively). Finally, it is interesting to note that among those whose identities changed, most did not give up their previous identities but rather incorporated additional ones. Also, 91% of the test-takers who were reinterviewed about 18 months later reported no change from their responses in the first interview. The most interesting conclusion from the interviews was that test-takers did not simply adopt what the tests results state. Rather, they made choices with the aim to embrace only those identities “that offer distinctiveness and provide social or psychological value”, while weighting the social cost of other rejecting or questioning these choices (Roth & Ivemark, 2018).

Roth and colleagues designed another study in order to test whether the results of ancestry tests increase essentialist views of race. Participants were randomly assigned to different groups in order to be able to distinguish between the effects of the test and the motivations of the test-takers. Without randomization, participants’ motivations (e.g., to prove their purity or admixture) could bias the findings. The study involved 802 native-born White people from the USA who were randomly assigned to one of two categories: half of them received Admixture and mtDNA tests purchased from *Family Tree DNA* and half received no test. These participants were asked questions that focused on what they thought about the relationship between genes and race, on a four-point (disagree/agree) response-scale (Yaylacı et al., 2019.) Overall, the results showed no significant effect of testing on essentialist views about race when comparing those who took the test and those who did not. However, when the researchers looked at the effect with respect to participants’ knowledge of genetics, there were differences. Among those who took a test, participants with high genetics knowledge showed a significant decline in their essentialist beliefs after taking the test. It is important to note that they already had lower genetic essentialism beliefs score before taking the ancestry test. In contrast, these beliefs increased among those with very limited knowledge of genetics; however, due to their limited numbers, the researchers suggested that this requires further research. There was also no change in essentialist beliefs with respect to participants’ specific ancestries. Therefore, a main conclusion was that participants’ genetics knowledge may be critical for how they understand the results of DNA ancestry tests and the inferences they make from it—but we should keep in mind that the study only included people who identified as White (Roth et al., 2020).

Speaking of White people, there is another study worth considering. Sociologist Aaron Panofsky and colleagues (2019) investigated how White nationalists, who by default hold a genetic essentialist view of race, interpret the results of DNA ancestry tests. There are several discussions about how White supremacists could “prove” their racial purity with DNA ancestry testing; and about how their opponents could rely on these tests to undermine their claims. What the researchers did was to analyze 639 posts from the white nationalist website Stormfront.org, posted between 2004 and 2016, in which users revealed the results of their DNA ancestry tests. The aim was to document the reactions of the community to results that did not confirm White racial purity. Among these 639 posts, it was possible for researchers to ascertain the testing technology used in 153 posts. In 51 of these, a detailed presentation of the results was made, without any identity aspiration. Among the remaining 102 posts, identity aspiration was confirmed in 53, with confirmation of prior knowledge occurring in 28 and a welcome surprise occurring in 25. In contrast, in the other 49 posts the users’ identity aspiration was challenged, with unwelcome surprises occurring in 28 posts, whereas in 21 posts uncertainty about how to interpret the results was expressed. The most important finding was that the reactions of the community were mostly about the interpretation of the tests’ results, rather than about the users posting them. The Stormfront community was generally devoted to address a user’s upsetting results, rather than criticizing them. This was done through rhetorical strategies that either rejected the results or reinterpreted them. Another interesting finding was that the community’s reactions depended more on the attitude of the user who made the announcement, than on the results themselves. Those users who appeared to be humble were more likely to receive helpful messages than those who were extremely defensive (Panovsky & Donovan, 2019).

## Conclusion

This paper draws a link between sociogenetic essentialism and the misinterpretation of DNA ancestry test results as affirming the existence of socially constructed categories such as race. Sociogenetic essentialism refers to the belief that human social groups have fixed and immutable essences in their DNA that can be used to categorize them. This premise contributes to people’s tendency to categorize others based on limited interactions or observations. The implications of such thinking extend to the perception of socially constructed categories, such as race and ethnicity, as natural. The use of ancestry DNA tests and the accompanying rhetoric have a strong potential to be interpreted in essentialist terms. Therefore, people may inaccurately magnify inferences about ethnicity, nationality, and race based on these tests, despite the tests’ primary purpose being to determine ancestry. Psychological essentialism is very pervasive and intuitive, and, as we have demonstrated above, it has a strong potential to affect the way people interpret ancestry test results, calling for a better understanding of this connection in order to nudge people effectively towards a more scientifically-aligned interpretation of ancestry data.

Importantly, the marketing of DNA ancestry testing may be interpreted in essentialist terms as well. Consider the following statement included in an advertisement by the company Ancestry, the largest DNA ancestry testing company: “You could be Irish. More specifically, Muster Irish”.^2^ This phrase might be interpreted as being based on particular essentialist assumptions:

I. That “Irish” or “Munster Irish” is a distinct group that can be delimited from others— otherwise there is no point in referring to such a group
II. That a person who is “Munster Irish” cannot also belong to another group, say being “Connacht Irish”— otherwise it is meaningless to say that one is “Munster Irish” if one could also be “Connacht Irish”
III. That there is something distinctive among “Munster Irish” people that makes them more similar to one another and more different from other categories, such as “Connacht Irish”— otherwise they could not be identified as such
IV. Being “Munster Irish” is something that we can find based on DNA—otherwise why should we do the DNA test anyway?— which means that DNA is the essence of who we are

So, even if this is entirely unintentional, psychological, sociogenetic essentialism might make people prone to essentialize social groups based on the results of DNA ancestry testing, even though these results are both tentative and relative (Kampourakis, 2023).

The combination of the lack of sufficient scientific understanding of genetics in the general non-expert population and the complexity of tracing and comprehending the assumptions at the basis of DNA ancestry test (e.g. the complex process of selecting a reference group for a particular region/category) result in ancestry-informative markers (AIMs) being misconstrued as affirming the existence of races/ethnicities. As DNA becomes increasingly prominent in the general discourse (ancestry testing, forensics, paternity testing etc.), it is arguable that the pitfalls of sociogenetic essentialism will become more salient. Given this potential, explicitly emphasizing ancestry metrics over essentialistic intuitions, such as race, and using language that reflects that emphasis may mitigate essentialism-based interpretations, reduce potential harms from misinterpretations, and promote accurate interpretation of ancestry test results.

## Footnotes

1 The first round of interviews was conducted between August 2009 and November 2010, whereas follow-up interviews with 89 respondents took place an average of 18 months after the first interview.

2 https://www.ancestry.com/dna/?oiid=109972&olid=109972&osch=Web+Property
